# Formation of large low shear velocity provinces through the decomposition of oxidized mantle

**DOI:** 10.1038/s41467-021-22185-1

**Published:** 2021-03-26

**Authors:** Wenzhong Wang, Jiachao Liu, Feng Zhu, Mingming Li, Susannah M. Dorfman, Jie Li, Zhongqing Wu

**Affiliations:** 1 Laboratory of Seismology and Physics of Earth’s Interior, School of Earth and Space Sciences, University of Science and Technology of China, Hefei, China; 2 Department of Earth Sciences, University College London, London, UK; 3 Department of Earth and Environmental Sciences, Michigan State University, East Lansing, MI USA; 4 Department of Earth and Environmental Sciences, University of Michigan, Ann Arbor, MI USA; 5 School of Earth and Space Exploration, Arizona State University, Tempe, AZ USA; 6 National Geophysical Observatory at Mengcheng, University of Science and Technology of China, Hefei, China; 7 CAS Center for Excellence in Comparative Planetology, USTC, Hefei, Anhui China

**Keywords:** Geodynamics, Geophysics, Mineralogy, Seismology

## Abstract

Large Low Shear Velocity Provinces (LLSVPs) in the lowermost mantle are key to understanding the chemical composition and thermal structure of the deep Earth, but their origins have long been debated. Bridgmanite, the most abundant lower-mantle mineral, can incorporate extensive amounts of iron (Fe) with effects on various geophysical properties. Here our high-pressure experiments and ab initio calculations reveal that a ferric-iron-rich bridgmanite coexists with an Fe-poor bridgmanite in the 90 mol% MgSiO_3_–10 mol% Fe_2_O_3_ system, rather than forming a homogeneous single phase. The Fe^3+^-rich bridgmanite has substantially lower velocities and a higher *V*_*P*_*/V*_*S*_ ratio than MgSiO_3_ bridgmanite under lowermost-mantle conditions. Our modeling shows that the enrichment of Fe^3+^-rich bridgmanite in a pyrolitic composition can explain the observed features of the LLSVPs. The presence of Fe^3+^-rich materials within LLSVPs may have profound effects on the deep reservoirs of redox-sensitive elements and their isotopes.

## Abstract (web summary)

Dense Fe^3+^-rich bridgmanite can explain the seismic features of Large Low Shear Velocity Provinces, as it can form large-scale thermochemical piles in the deep mantle that remain stable throughout Earth’s history.

## Introduction

The large low shear velocity provinces (LLSVPs) are two massive and mysterious regions sitting beneath Africa and the Pacific^1–3^ and occupy ~3–9% of the volume of the Earth^3,4^. They are characterized by their lower-than-average seismic wave velocities^4^ and extend by thousands of kilometers laterally and up to >1000 km vertically above the core–mantle boundary (CMB)^3,4^. As the largest seismic heterogeneities in the lower mantle, they may hold the key to understanding the thermal, chemical, and dynamical evolution of the Earth^5,6,7^. Different shear-wave tomography models^4^ have reached agreement that the shear-wave velocity anomaly (dln*V*_*S*_) ranges from −0.5 to −1.0% in the shallow part of LLSVPs, while it could be up to −3.0% in the bottom part^6^. The compressional-wave tomography models also reveal negative anomalies of compressional-wave velocities (*V*_*P*_) (refs. ^8,9^), although the amplitude, shape, and geographical location of *V*_*P*_ anomaly vary widely among different models^5^. The *V*_*P*_ anomaly generally has a smaller amplitude than the *V*_*S*_ anomaly, causing a high dln*V*_*S*_/dln*V*_*P*_ ratio^9^. In particular, waveform and travel-time seismic studies^1,3,10–12^ reveal that the LLSVPs have sharp edges along their margins, which is also supported by the large lateral d*V*_*S*_ gradients at the boundary of LLSVPs^6^.

The systematic and discontinuous contrasts in seismic properties indicate that the LLVSPs are likely composed of distinct chemical materials from the surrounding mantle^11,13^. A chemically distinct origin of the LLSVPs may be implicated by their density anomalies as well; however, large discrepancies for the density anomaly associated with the LLSVPs exist in the literature^5^. Recent tidal tomography based on body tide displacements^14^ found that the mean density of the lower two-thirds of the two LLSVPs is ~0.5% higher than that of the surrounding mantle. On the contrary, a study using Stoneley modes suggested an overall negative density anomaly within LLSVPs, without excluding the possibility of a high-density anomaly within the lowermost LLSVPs^15^. It is still unknown whether the regional differences in density anomaly are caused by the choice of observations used to constrain density models or reflect the nature of LLSVPs associated with their origins.

Hypotheses for the origin of chemically distinct LLSVPs include processes associated with the accumulation of subducted oceanic crust over Earth history^16^ and the differentiation and solidification of an ancient basal magma ocean^6^. Sunken piles of subducted oceanic crust, which is compositionally different from and significantly denser than the pyrolitic lower mantle^17^, was proposed to explain LLSVPs because of the low velocity of calcium silicate perovskite (CaSiO_3_, CaPv) (ref. ^18^). However, there are significant discrepancies in the velocity of CaPv between two experimental studies^18,19^ and between experiments and theoretical results^20^. Elastic properties from ab initio simulations for the entire MORB assemblage indicate that subducted oceanic crust has relatively higher velocities than the ambient mantle^21^. Moreover, geodynamic simulations^22^ suggested that the present-day subducted oceanic crust is too thin to provide enough negative buoyancy to ﻿survive viscous stirring and it hence is difficult to amass coherent thermochemical structures and shapes at the CMB similar to LLSVPs^23^. Alternatively, LLSVPs may be composed of primordial residues from basal magma ocean crystallization or core–mantle differentiation that have not yet been fully homogenized by the mantle convection^24–26^. These primordial materials would need to be intrinsically more dense than the surrounding mantle to overcome mantle stirring^27^. Dense Fe–Ni–S liquid, for instance, was proposed to explain the LLSVPs^28^, but the amount of this liquid remaining in the deep mantle, which depends on the drainage of melt to the core^29^, is under debate.

Consistent with both efficient drainage of metallic melt and a primordial origin of the LLSVPs is chemical heterogeneity produced by redox reactions in the magma ocean. Ferrous iron (Fe^2+^) in silicate melts has been observed to disproportionate to ferric iron (Fe^3+^) plus metallic iron (Fe^0^) at high pressures^30^. Segregation of precipitated Fe^0^ from the magma ocean into the core would enrich Fe^3+^ in the mantle. Bridgmanite (Bdg), the dominant Fe^3+^-bearing mantle mineral, hosts Fe^3+^ through the Fe^3+^–Fe^3+^ or Fe^3+^–Al^3+^ charge-coupled substitution in the deep mantle^31–35^. In particular, a Fe^3+^-rich Bdg with the chemical composition of (Mg_0.5_Fe_0.5_)(Si_0.5_Fe_0.5_)O_3_ was recently synthesized by Liu et al.^36^. Oxidized domains enriched with such Fe^3+^-rich Bdg would be distinct from a pyrolitic lower mantle in sound velocity and density^37,38^ and may be responsible for the origins of lower-mantle seismic and geochemical heterogeneities, such as the LLSVPs^1–3,6^. The conditions of formation of such Fe^3+^-rich Bdg in a mantle phase assemblage and its elastic properties at lower-mantle-relevant temperatures are vital to test this hypothesis, but these questions remain unclear.

In this work, we combine high pressure-temperature (*P-T*) experiments, ab initio calculations, and geodynamic simulations to study the formation of Fe^3+^-rich Bdg, its thermoelastic properties, and its dynamics in the mantle, to evaluate whether enrichment in Fe^3+^-rich Bdg can explain the seismic signatures of LLSVPs.

## Results

### The coexistence of Fe^3+^ -rich and Fe-poor bridgmanite phases

**Fig. 1:**
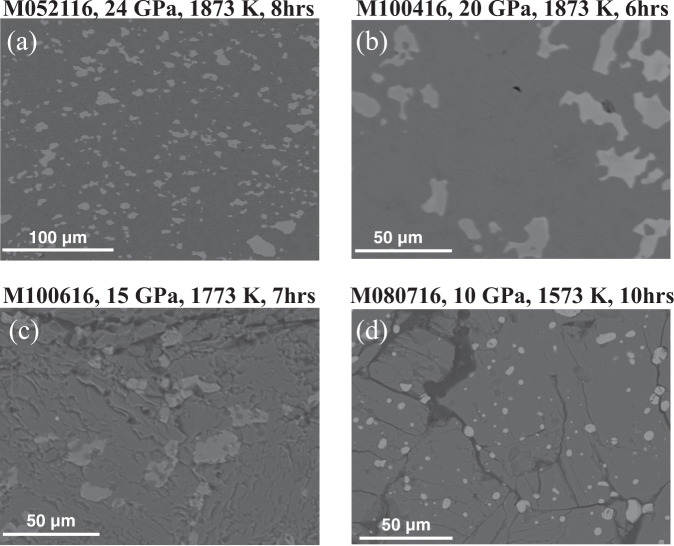
Back-scattered scanning electron microscope images of recovered experimental phase assemblages forming from 90% MgSiO_3_ +  10% Fe_2_O_3_ starting mixture after equilibrium at mantle pressure–temperature conditions for 6–10 h. Bridgmanite/akimotoite with ~50 mol% Fe_2_O_3_ substitution in both Mg and Si sites (bright phases in (**a**–**c**)) coexists with normal mantle minerals such as Fe-poor bridgmanite (dark phases in (**a**) and (**b**)) at >20 GPa, or with wadsleyite (gray phase in (**c**)) + stishovite (dark phase in (**c**)) at 15 GPa, while Fe_2_O_3_ (bright phase in (**d**)) was stabilized as a separate phase with Fe-depleted clinopyroxene (dark phase in (**d**)) at 10 GPa.

We conducted a series of multi-anvil experiments with the bulk composition of 90 mol% MgSiO_3_–10 mol% Fe_2_O_**3**_ from 10-24 GPa along a mantle geotherm (Supplementary Table 1). At 10 GPa and 1573 K (Fig. 1d), the run products consist of separate MgSiO_3_ and Fe_2_O_3_ phases, demonstrating low solubility of Fe_2_O_3_ in clinopyroxenes at upper-mantle conditions due to the incompatibility between large Fe^3+^ and small tetrahedral site. At 15 GPa, an iron-rich akimotoite (Aki) forms with 33 mol% of MgSiO_3_ and 67 mol% of Fe_2_O_3_, coexisting with (Mg_1.79_Fe_0.18_)SiO_4_ wadsleyite and SiO_2_ stishovite (Fig. 1c). This indicates that the iron-rich Aki coexisting with iron-depleted oxides/silicates are energetically more stable than a single-phase MgSiO_3_–Fe_2_O_3_ solid solution with intermediate iron content. At 24 GPa and 1873 K, which corresponds to the *P–T* conditions around the uppermost lower mantle, the products consist of Fe-poor Bdg and Fe-rich Aki with 44–49 mol% Fe in both Mg and Si sites (Fig. 1a and Supplementary Table 1), instead of forming a single phase of (Mg_0.9_Fe_0.1_)(Si_0.9_Fe_0.1_)O_3_ Bdg. The Fe-rich Aki is evenly distributed in the matrix of the Fe-poor Bdg (Fig. 1a). The run products of experiments running at the same *P–T* conditions for 8 and 24 h have the same compositions within analytical uncertainty (Supplementary Table 1), confirming that the experiments reached equilibrium. In situ XRD measurements coupled with a diamond anvil cell (DAC) show that this Fe-rich Aki phase transforms to Bdg phase at 23.5 ± 1.0 GPa and 300 K (Supplementary Fig. 1). Moreover, this Fe^3+^-rich Bdg phase completely transforms back to Aki with the same lattice parameters as the starting Aki phase after the decompression of the DAC (Supplementary Fig. 1). The reversible phase transition of this Fe-rich phase means that for our multi-anvil experiments at the *P–T* conditions of the uppermost lower mantle, Fe^3+^-rich Bdg coexists with Fe-pool Bdg.

A previous multi-anvil study^39^ synthesized Fe^3+^-only bridgmanite with 2–4 mol% Fe^3+^ in the cation sites but did not observe the Fe^3+^-rich Bdg phase, possibly because the bulk Fe content of their experiments is not high enough to enable the formation of such Fe^3+^-rich Bdg. Moreover, the presence of unreacted MgO and SiO_2_ in their run products (Fig. 1 in ref. ^39^) suggests that their starting materials may not be homogenous or their experiments did not reach chemical equilibrium. Another study^40^ synthesized Fe^3+^-only Bdg with the starting material of 90 mol% MgSiO_3_–10 mol% Fe_2_O_3_ using laser-heated diamond anvil cell (LH-DAC)^40^. However, the chemical composition of Fe^3+^-only Bdg was not reported, possibly because there was a significant loss of Fe and Mg during melting^40^, and some Fe^3+^ was reduced through reaction with diamond during laser heating^41^. In addition, the proportion of the Fe-rich Bdg phase is much smaller than the Fe-poor Bdg (Fig. 1), and therefore it is difficult to detect without a detailed analysis of the run products in ref. ^40^.

We also performed ab initio calculations (see methods and supplementary materials) to investigate the stability of (Mg_0.5_Fe_0.5_)(Si_0.5_Fe_0.5_)O_3_ Bdg under lower-mantle conditions. Our results show that the assemblage of (Mg_0.5_Fe_0.5_)(Si_0.5_Fe_0.5_)O_3_ and MgSiO_3_ Bdg has a lower Gibbs free energy than a single-phase (Mg_0.875_Fe_0.125_)(Si_0.875_Fe_0.125_)O_3_ Bdg under the *P–T* of the whole lower mantle regardless of the spin state (Supplementary Fig. 2), indicating that the mixed two phases are more stable than the single-phase Bdg with a homogeneous composition. The theoretical results support our experimental observations and reveal that this Fe^3+^-rich Bdg with the chemical composition of approximately (Mg_0.5_Fe_0.5_)(Si_0.5_Fe_0.5_)O_3_ should form as a separate phase coexisting with Fe-poor Bdg in the bulk composition of 90 mol% MgSiO_3_–10 mol% Fe_2_O_3_ due to the miscibility gap.

### Elastic properties and sound velocities of (Mg_0.5_Fe_0.5_)(Si_0.5_Fe_0.5_)O_3_ bridgmanite

**Fig. 2:**
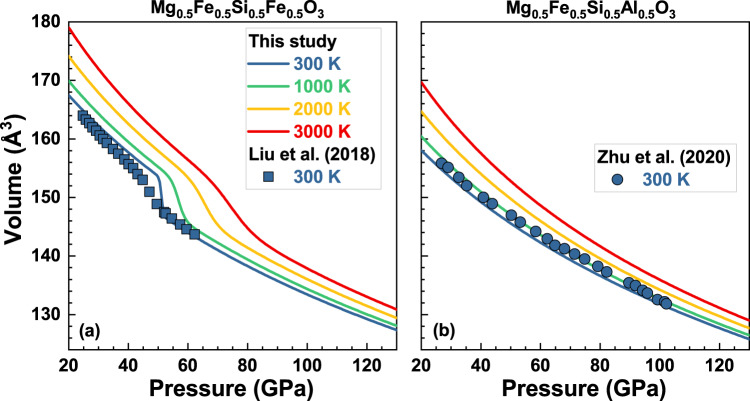
Isothermal compression curves predicted by ab initio calculations for (Mg_0.5_Fe_0.5_)(Si_0.5_Fe_0.5_)O_3_ and (Mg_0.5_Fe_0.5_)(Si_0.5_Al_0.5_)O_3_ bridgmanite. **a** (Mg_0.5_Fe_0.5_)(Si_0.5_Fe_0.5_)O_3_ bridgmanite, **b** (Mg_0.5_Fe_0.5_)(Si_0.5_Al_0.5_)O_3_ bridgmanite. The blue, green, orange, and red curves are calculated compression curves of (Mg_0.5_Fe_0.5_)(Si_0.5_Fe_0.5_)O_3_ and (Mg_0.5_Fe_0.5_)(Si_0.5_Al_0.5_)O_3_ bridgmanite at 300, 1000, 2000, and 3000 K, respectively. The blue squares are experimental measurements from Liu et al.^36^, which shows that the spin transition of Fe^3+^ in the B-site of (Mg_0.5_Fe_0.5_)(Si_0.5_Fe_0.5_)O_3_ bridgmanite occurs between 43 and 53 GPa at 300 K. The blue circles are experimental results of Zhu et al.^42^.

Determining the seismic signature of a separate Fe^3+^-rich phase in equilibrium with the mantle phase assemblage requires elastic properties of this phase as a function of pressure and temperature conditions in the mantle. The elastic properties of (Mg_0.5_Fe_0.5_)(Si_0.5_Fe_0.5_)O_3_ Bdg up to 130 GPa and 3000 K were theoretically obtained from ab initio calculations. Because Bdg may also accommodate Al in the octahedral site^31,38,42^, we also conducted calculations on an end-member composition (Mg_0.5_Fe_0.5_)(Si_0.5_Al_0.5_)O_3_, to quantify the effect of Al on the elasticity of Bdg. In these calculations, the octahedral site (B-site) Fe^3+^ in (Mg_0.5_Fe_0.5_)(Si_0.5_Fe_0.5_)O_3_ Bdg undergoes a high-spin (HS) state to a low-spin (LS) state transition with increasing pressure (Fig. 2a), while the dodecahedral-site (A-site) Fe^3+^ in both compositions remain in the HS state throughout the lower-mantle conditions^43^.

Our calculated volumes of HS- and LS-(Mg_0.5_Fe_0.5_)(Si_0.5_Fe_0.5_)O_3_ Bdg agree well with experimental measurements at 300 K (ref. ^36^) (Fig. 2a). The calculated spin transition of the B-site Fe^3+^ occurs between 49 and 55 GPa at 300 K^36^, which is slightly higher and narrower than experimental results^36^. The predicted ﻿volume collapse (Δ*V*^HS-LS^) caused by the spin transition of B-site Fe^3+^ is ~4.3% at 300 K, higher than experimental measurements (2.7%)^36^ but consistent with previous theoretical calculations^44^ on (Mg_0.5_Fe_0.125_)(Si_0.5_Fe_0.125_)O_3_ Bdg assuming that Δ*V*^HS-LS^ is linearly dependent on Fe^3+^ content. The Δ*V*^HS-LS^ discrepancy between experimental and theoretical studies is probably caused by the difference in the pressure range for the mixed-spin (MS) state. The predicted volumes of (Mg_0.5_Fe_0.5_)(Si_0.5_Al_0.5_)O_3_ Bdg also show excellent agreement with experimental results at 300 K^42^. These comparisons demonstrate the high reliability of our DFT + *U* calculations in predicting elastic properties, as suggested by previous studies^43–45^.

The spin transition of B-site Fe^3+^ in (Mg_0.5_Fe_0.5_)(Si_0.5_Fe_0.5_)O_3_ Bdg generates a strong effect on bulk modulus (*K*_*S*_) and *V*_*P*_, which both show deep valleys that broaden and decrease in magnitude with increasing temperature (Supplementary Fig. 3). The magnitude and width of the *K*_*S*_ and *V*_*P*_ anomalies are controlled by the fraction of LS B-site Fe^3+^ (n_LS_) and the pressure and temperature dependences of n_LS_ (see Supplementary Materials). Compared to MgSiO_3_ Bdg^46^, both (Mg_0.5_Fe_0.5_)(Si_0.5_Fe_0.5_)O_3_ and (Mg_0.5_Fe_0.5_)(Si_0.5_Al_0.5_)O_3_ Bdg have much lower elastic moduli (*K*_*S*_ and *G*) and velocities (*V*_*P*_ and *V*_*S*_) (Supplementary Fig. 4). At lowermost-mantle conditions, the differences in *K*_*S*_, *G, V*_*P*_, and *V*_*S*_ between (Mg_0.5_Fe_0.5_)(Si_0.5_Fe_0.5_)O_3_ and MgSiO_3_ Bdg^46^ are about −5%, −37%, −17%, and −28%, respectively, which in turn causes a higher *V*_*P*_*/V*_*S*_ ratio of 2.1 in (Mg_0.5_Fe_0.5_)(Si_0.5_Fe_0.5_)O_3_ Bdg (Supplementary Fig. 4). By comparison, the differences in *K*_*S*_, *G, V*_*P*_, and *V*_*S*_ between (Mg_0.5_Fe_0.5_)(Si_0.5_Al_0.5_)O_3_ and MgSiO_3_ Bdg are about −4%, −18%, −9%, and −14%, respectively.

## Discussion

**Fig. 3:**
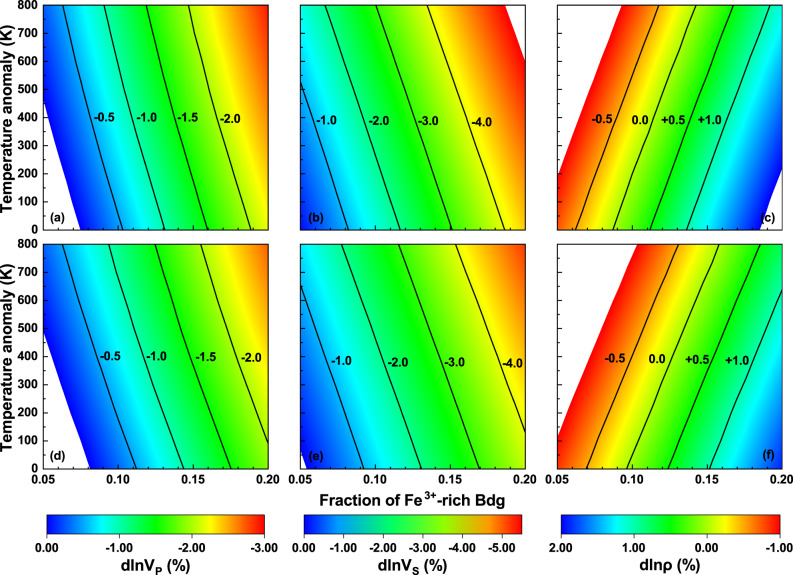
Velocity and density anomalies caused by the enrichment of Fe^3+^-rich bridgmanite relative to pyrolitic composition. **a**, **d** V_P_, **b**, **e** V_S_, and **c**, **f** density anomalies at 110 GPa. **a**–**c** Anomalies due to enrichment of (Mg_0.5_Fe_0.5_)(Si_0.5_Fe_0.5_)O_3_ bridgmanite (Bdg); **d**–**f** anomalies due to enrichment of (Mg_0.5_Fe_0.5_)(Si_0.5_Fe_0.4_Al_0.1_)O_3_ Bdg, where 5% Al_2_O_3_ is incorporated into (Mg_0.5_Fe_0.5_)(Si_0.5_Fe_0.5_)O_3_. Velocities and density of the pyrolitic lower mantle are calculated using the best-fit composition of the lower mantle (15% Mg_0.82_Fe_0.18_O ferropericlase (Fp), 78% Mg_0.92_Fe_0.08_SiO_3_ bridgmanite (Fe^2+^-Bdg), and 7% CaSiO_3_ Ca-perovskite (CaPv))^37^. The modeling chemical assemblage has pyrolitic mineral fractions in which a portion of Fe^2+^-bearing Bdg was substituted by (Mg_0.5_Fe_0.5_)(Si_0.5_Fe_0.5_)O_3_ or (Mg_0.5_Fe_0.5_)(Si_0.5_Fe_0.4_Al_0.1_)O_3_ Bdg. The initial Fe^2+^O contents of Bdg and Fp in the modeling assemblage are 4 and 9 mol%, respectively, which are half of those in the reference pyrolite^37^. The temperature anomaly is with respect to the normal mantle temperature from Brown and Shankland^74^. Data for elasticity at high pressure and temperature are derived from previous theoretical studies: Fp, ref. ^47^; Fe^2+^-Bdg, ref. ^46^; CaPv, ref. ^20^.

Combining elastic data from previous studies^20,45–47^ with our results, we modeled the density and velocity anomalies caused by the presence of Fe^3+^-rich Bdg relative to the pyrolitic composition, which can effectively reproduce the reference seismic velocities and density of PREM^37,38^. The modeled chemical assemblage has pyrolitic mineral fractions (15% ferropericlase (Fp) + 78% Bdg + 7% CaPv) in which a portion of Fe^2+^-bearing Bdg was substituted by (Mg_0.5_Fe_0.5_)(Si_0.5_Fe_0.5_)O_3_ Bdg. We find that the enrichment of (Mg_0.5_Fe_0.5_)(Si_0.5_Fe_0.5_)O_3_ Bdg in the assemblage can explain the seismic features of the LLSVPs^4,6,8,9^. The *V*_*S*_ anomalies of −1.5% to −3.0% and the large dln*V*_*S*_/dln*V*_*P*_ ratio >2.0 observed in LLSVPs^4,6,8,9^ can be reproduced by the enrichment of 10–15% (Mg_0.5_Fe_0.5_)(Si_0.5_Fe_0.5_)O_3_ Bdg in pyrolite at 110 GPa (Fig. 3). If LLSVPs are hotter (Δ*T*_LLSVPS_ > 0), the required proportion of (Mg_0.5_Fe_0.5_)(Si_0.5_Fe_0.5_)O_3_ Bdg would accordingly decrease; for example, it will decrease by ~2% if Δ*T*_LLSVPS_ is +400 K. For a pyrolitic composition, Bdg also contains ~5 mol% Al_2_O_3_, which does not significantly change its velocities and density^48^. When 5 mol% Al_2_O_3_ is incorporated into Bdg, the *V*_*P*_ and *V*_*S*_ anomalies caused by the presence of 15% (Mg_0.5_Fe_0.5_)(Si_0.5_Fe_0.4_Al_0.1_)O_3_ Bdg at Δ*T*_LLSVPS_ equal to +400 K will be −1.5% and −3.1% (Fig. 3), respectively, which can also reproduce the dln*V*_*S*_/dln*V*_*P*_ ratio >2.0.

**Fig. 4:**
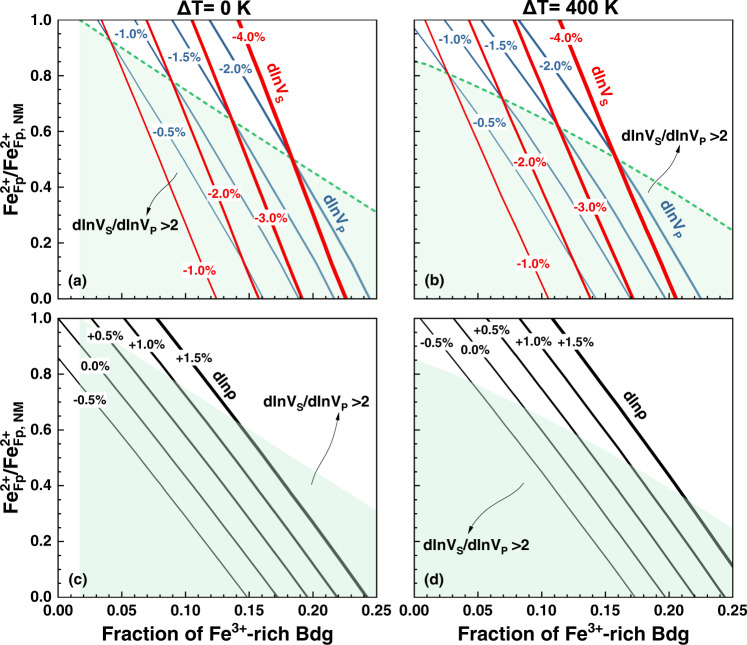
Velocity and density anomalies versus Fe^2+^ content and fraction of (Mg_0.5_Fe_0.5_)(Si_0.5_Fe_0.5_)O_3_ bridgmanite. **a**, **b** V_P_ and V_S_ anomalies (solid blue and red lines, respectively); **c**, **d** density perturbation (solid black lines). The line thickness refers to the magnitude as noted by the number. **a**, **c** Temperature anomaly is 0 K *(*Δ*T*_LLSVPS_ = 0 K); **b**, **d** Δ*T*_LLSVPS_ = +400 K. The pyrolitic lower mantle is composed of 15% Mg_0.82_Fe_0.18_O Fp, 78% Mg_0.92_Fe_0.08_SiO_3_ bridgmanite (Bdg), and 7% CaSiO_3_ CaPv)^37^. Fe^2+^_Fp, NM_ refers to the FeO content of Fp (18 mol%) in the pyrolite model for the normal lower mantle, and the Fe–Mg partition coefficient between Fp and Bdg^73^ is used to constrain their Fe^2+^ contents. The modeled assemblage with different Fe^2+^_Fp_/Fe^2+^_Fp, NM_ ratios has identical mineral fractions to pyrolitic composition in which a portion of Fe^2+^-bearing Bdg was substituted by (Mg_0.5_Fe_0.5_)(Si_0.5_Fe_0.5_)O_3_ Bdg. Dash green lines represent the dln*V*_*S*_/dln*V*_*P*_ ratio of 2.0 and the light green shadows refer to compositional spaces that can reproduce the large dln*V*_*S*_/dln*V*_*P*_ ratio >2.0.

In addition, we find that the required proportion of (Mg_0.5_Fe_0.5_)(Si_0.5_Fe_0.5_)O_3_ Bdg increases with the decreasing of Fe^2+^ content in the modeled assemblage (noted by the FeO content in Fp, Fe^2+^_Fp_) to explain the same *V*_*P*_ and *V*_*S*_ anomalies (Fig. 4). If there is no Fe^2+^, the (Mg_0.5_Fe_0.5_)(Si_0.5_Fe_0.5_)O_3_ fraction that can reproduce the *V*_*S*_ anomaly of −3.0% would be raised to ~17% at Δ*T*_*LLSVPS*_ equal to +400 K (Fig. 4 and Supplementary Fig. 5), which corresponds to a *V*_*P*_ anomaly of ~−1.0% and a dln*V*_*S*_/dln*V*_*P*_ ratio of ~3.0. The dln*V*_*S*_/dln*V*_*P*_ ratio >2.0 tends to be reproduced at relatively low Fe^2+^ contents (Fig. 4). If Fe^2+^_Fp_/Fe^2+^_Fp, NM_ > 0.9 (Fe^2+^_Fp, NM_ is the FeO content of Fp in a normal pyrolitic lower mantle, 18 mol%), the dln*V*_*S*_/dln*V*_*P*_ ratio is less than 2.0. In contrast, when Fe^2+^_Fp_/Fe^2+^_Fp, NM_ is <0.25, the dln*V*_*S*_/dln*V*_*P*_ ratio >2.0 can be reproduced for different *V*_*S*_ anomalies (Fig. 4). Our modeling implies that the enrichment of (Mg_0.5_Fe_0.5_)(Si_0.5_Fe_0.5_)O_3_ Bdg in the pyrolite assemblage with relatively lower Fe^2+^ content can explain the velocity anomalies and the dln*V*_*S*_/dln*V*_*P*_ ratio observed in LLSVPs^4,6,8,9^.

In contrast to velocity anomalies, the modeled density anomaly (dln*ρ*) could be positive, zero, or negative, depending on the (Mg_0.5_Fe_0.5_)(Si_0.5_Fe_0.5_)O_3_ abundance, temperature anomaly, and the fraction of Fe^2+^ in Fp. To produce a *V*_*S*_ anomaly of −3.0% for the assemblage enriched in (Mg_0.5_Fe_0.5_)(Si_0.5_Fe_0.5_)O_3_ Bdg, the dln*ρ* decreases from +1.2% at Δ*T*_LLSVPS_ of 0 K and Fe^2+^_Fp_/Fe^2+^_Fp, NM_ of 0.5 to −0.5% at Δ*T*_LLSVPS_ of +400 K and Fe^2+^_Fp_/Fe^2+^_Fp, NM_ of 0.0 (Figs. 3, 4 and Supplementary Fig. 5). In general, the density anomaly is correlated with the magnitude of *V*_*S*_ anomalies. For dln*V*_*S*_ < −1.0%, the dln*ρ* could be positive if Δ*T*_LLSVPS_ is 0 K; however, if Δ*T*_LLSVPS_ equals to +400 K, the dlnρ could be positive only when dln*V*_*S*_ is <−2.5% (Fig. 4). Our modeling suggests that the lowermost parts of LLSVPs with large negative velocity anomalies (<−3.0%)^4,6,8^ could be denser than the ambient mantle, while the relatively shallow part of LLSVPs with −0.5 to −1.0% *V*_*S*_ anomalies on average likely have slightly lighter density than the ambient mantle. Recent work proposed that the bottom two-thirds of the two LLSVPs are ~0.5% denser than the surrounding mantle^14^, while Koelemeijer et al.^15^ argued that the overall density of the LLSVPs is lower than the surrounding mantle. Such different conclusions may be related to different depth sensitivities of the datasets considered^49^, regardless of the input observations. Also, geodynamic modeling studies^50,51^ suggested that the density anomalies of the LLSVPs relative to the surrounding mantle could be positive near the top and bottom of the LLSVPs but neutral or slightly negative in the middle of the LLSVPs. The density of LLSVPs could also be laterally inhomogeneous due to their internal convection and the entrainment of multiple compositional components into the LLSVPs^52^.

**Fig. 5:**
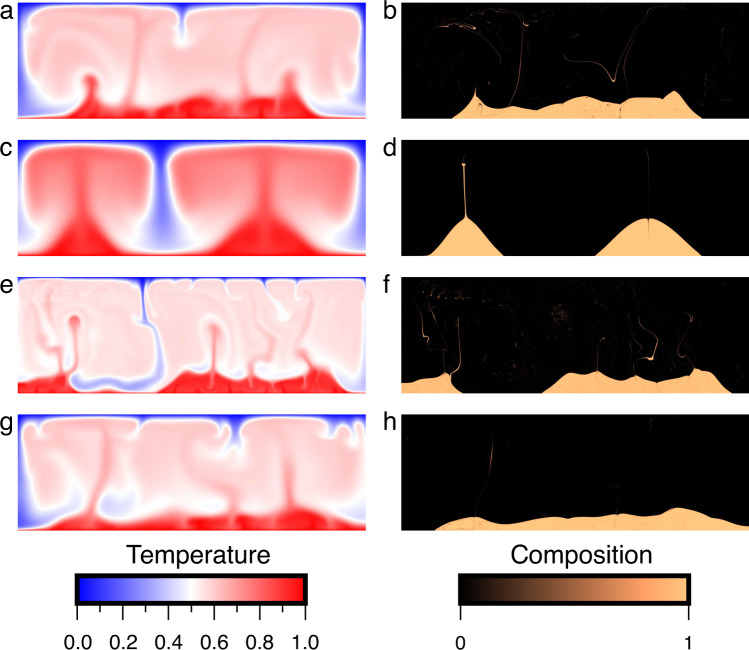
Formation of thermochemical piles in the lowermost mantle from iron-rich materials. The temperature (left column) and composition (right column) fields from the surface to the core–mantle boundary are shown at 4.5 Gyr for case 1 (**a**, **b**), case 2 (**c**, **d**), case 3 (**e**, **f**), and case 4 (**g**, **h**). In cases 1, 2, 3, and 4, the Rayleigh number is *Ra* = 1 × 10^7^, 1 × 10^6^, 1 × 10^8^, and 1 × 10^7^, respectively, and the iron-rich materials (shown by golden colors in the right panels) are 1.2%, 1.2%, 1.2%, and 1.5% intrinsically denser than the background mantle materials (shown by black colors in the right panels), respectively. Large thermochemical piles form after 4.5 Gyr for all cases.

The present model for chemical heterogeneities within the LLSVPs is consistent with Fe-rich remnants of a basal magma ocean created early in Earth’s history^6,24–26^. Ferrous Fe in dense silicate melts associated with the basal magma ocean would partially disproportionate to Fe^3+^ plus Fe^0^ at high pressures^30^ and segregation of precipitated Fe^0^ from the magma ocean into core would enrich silicate melt in Fe_2_O_3_ component. A thermodynamic model of magma ocean crystallization^53^ suggests that the silicate melt fraction would be gradually enriched in iron with Fe/(Fe+Mg) >0.3 in the lower mantle after 60 wt% of the melt has solidified. The Fe/(Fe+Mg) ratio in the residual melt remaining in the lowermost mantle could be up to 0.5 near the end of the crystallization. The amount of Fe^3+^ in this melt depends on the amount of Fe^2+^ that would disproportionate into Fe^3+^ plus Fe^0^ and the efficiency of Fe^0^ droplet segregation. The required bulk composition with MgSiO_3_:Fe_2_O_3_ equal to 9:1 could be produced when 40–80% Fe^2+^ undergoes the disproportionation reaction and all Fe^0^ migrates into the core. Fe^3+^ would be incorporated into bridgmanite with further crystallization, and our experiments and ab initio calculations indicate that in these Fe^3+^-rich regions, a portion of (Mg_0.5_Fe_0.5_)(Si_0.5_Fe_0.5_)O_3_ silicate would form as a separate phase, coexisting with Fe^3+^-poor silicate. Due to the large excess density, (Mg_0.5_Fe_0.5_)(Si_0.5_Fe_0.5_)O_3_ silicate could descend to the base of the lower mantle through mantle convection and result in Fe^3+^-rich bridgmanite piles. Our geodynamic modeling demonstrates that such Fe^3+^-rich piles with ~18% (Mg_0.5_Fe_0.5_)(Si_0.5_Fe_0.5_)O_3_ Bdg, which is ~1.5% intrinsically denser than the ambient mantle (Fig. 4c), could form large-scale thermochemical structures in the lowermost mantle without being mixed into the background mantle throughout Earth’s history (Fig. 5), which may accumulate to form LLSVPs.

Our findings imply that the segregation of Fe^3+^-rich domains may cause heterogeneity in the redox states of the Earth’s mantle, that is, the oxygen fugacity of the lowermost mantle may not be as low as inferred in previous studies^54,55^. We also expect these Fe^3+^-rich domains to be enriched in heavy iron isotopes because Fe^3+^ has a larger Fe force constant than Fe^2+^ (ref. ^56^), which may affect the iron isotopic features of the deep Earth^57^. The presence of lower-mantle oxidizing heterogeneities would have profound effects on the cycles of volatiles^30^ and the deep reservoirs of redox-sensitive elements. For instance, the dense reduced Fe-C/H/S melts formed at the mantle transition zone and shallow lower mantle by slab/mantle interaction^58,59^, if they reach the lowermost mantle, could be converted back to an oxidized state instead of sinking into the core. Dynamic cycling with respect to mantle redox heterogeneity could provide new insights into the thermochemical evolution of the bulk silicate Earth and possibly the oxidation of the atmosphere.

## Methods

### High pressure–temperature experiments

The experiments were conducted using the 1000-ton multi-anvil apparatus at the University of Michigan. The COMPRES 8/3 and 10/5 cell assemblies were employed in the experiments. The starting material was a mixture of high purity (>99.99%) MgO, SiO_2_, and Fe_2_O_3_ at a molar ratio of 9:9:1, which corresponds to a bulk composition of (Mg_0.9_Fe_0.1_)(Si_0.9_ Fe_0.1_)O_3_. The mixture was heated at 1073 K overnight to remove the moisture and structural water before loading into a platinum capsule. The sample was compressed to target pressure and equilibrated at high temperature for 6–10 h to allow sufficient equilibrium. It was then quenched to room temperature and decompressed to 1 bar.

The recovered sample was polished, coated with carbon, and examined for texture and composition using the JOEL-7800FLV Scanning Electron Microprobe (SEM) and SX-100 Electron Microprobe Analysis (EPMA) at the Electron Microbeam Analysis Laboratory (EMAL) of the University of Michigan. An accelerating voltage of 15 kV and a beam current of 10 nA were employed for imaging and analysis. Forsterite and magnetite were used as standards for Mg, Si, and Fe quantification with EPMA.

### First-principles calculations

Isothermal elastic tensors ($C_{ijkl}^T$) of crystals in a Cartesian coordinate system usually can be calculated from Eq. (1) (ref. ^60^):

1 $$C_{ijkl}^T = \frac{1}{V}(\frac{{\partial ^2F}}{{\partial e_{ij}\partial e_{kl}}}) + \frac{1}{2}P(2\delta _{ij}\delta _{kl} - \delta _{il}\delta _{kj} - \delta _{ik}\delta _{jl})$$

where e_*ij*_(*i,j* = 1,3) are infinitesimal strains, *P* is the isotropic pressure, and *F* is the Helmholtz free energy, which can be expressed in the quasi-harmonic approximation (QHA) as:

2 $$F\left( {e_{ij},V,T} \right) = U\left( {e_{ij},V} \right) + \frac{1}{2}\mathop {\sum }\limits_{q,m} \hbar \omega _{q,m}\left( {e_{ij},V} \right) + k_BT\mathop {\sum }\limits_{q,m} ln(1 - {\mathrm{exp}}( - \frac{{\hbar \omega _{q,m}\left( {e_{ij},V} \right)}}{{k_BT}}))$$

where *V* is the equilibrium volume of the crystal and *T* is temperature. Subscripts *q* and *m* refer to the phonon wave vector and the normal mode index, respectively. $\hbar$ and *k*_*B*_ are Planck and Boltzmann constants, and *ω*_*q,m*_ is the vibrational frequency of the *i*th mode along with the wave vector *q*. *U* is the static energy at the equilibrium volume *V*. The second and third terms are the zero-point and vibrational energy contributions, respectively. Adiabatic elastic constants ($C_{ijkl}^S$) can be derived from:

3 $$C_{ijkl}^S = C_{ijkl}^T + \frac{T}{{VC_V}}\frac{{\partial S}}{{\partial e_{ij}}}\frac{{\partial S}}{{\partial e_{kl}}}\delta _{ij}\delta _{kl}$$

where *S* is the entropy and *C*_*V*_ is the constant volume heat capacity. Therefore, calculations of elastic tensors at high pressure and temperature using this usual method require a vibrational density of states (VDoS) of many strained configurations, which demand a tremendous amount of computational power to calculate based on the DFT. Wu and Wentzcovitch^61^ proposed an analytical approach to calculate the thermal contribution to the elastic tensor, only requiring VDoS for unstrained configurations at different equilibrium volumes. This approach greatly reduces the computation cost to the level of <10% of the usual method without loss of accuracy.

To obtain elastic tensors at static conditions and VDoS for unstrained configurations at different equilibrium volumes, we performed first-principles calculations using Quantum Espresso package^62^ based on the DFT, plane wave, and pseudopotential. Local density approximation (LDA) was adopted for the exchange-correlation function. The energy cutoff for electronic wave functions was set as 70 Ry. The Mg pseudopotential was generated using the von Barth and Car method for all channels using a 2.5 Bohr cutoff radius and five configurations, 3s^2^3p^0^, 3s^1^3p^1^, 3s^1^3p^0.5^3d^0.5^, 3s^1^3p^0.5^, 3s^1^3d^1^, with weights of 1.5, 0.6, 0.3, 0.3, 0.2, respectively. The pseudopotentials for Si and O were generated using the Troullier-Martins method^63^ with the cutoff radius of 1.47 Bohr for Si and 1.45 Bohr for O. Valence configurations for Si and O are 3s^2^3p^4^3d^0^ and 2s^2^2p^4^, respectively. The pseudopotentials for Al and Fe were generated using the Vanderbilt method^64^ with a valence configuration of 3s^2^3p^1^ and a cutoff radius of 1.77 Bohr for Al, and a valence configuration of 3s^2^3p^6^3d^6.5^4s^1^4p^0^ and a cutoff radius of 1.8 Bohr for Fe. To address the large on-site Coulomb interactions among the localized electrons (Fe 3d electrons)^65^, we introduced a Hubbard *U* correction to the LDA (LDA+*U*) for all DFT calculations. *U* values for Fe^3+^ on A and B sites in bridgmanite were non-empirically determined using linear response method^66^ in previous work^43^ and adopted in this study. The initial structure was constructed by replacing one nearest-neighbor Mg^2+^–Si^4+^ pair with one [Fe^3+^]_Mg_–[Fe^3+^]^Si^ pair^43–45^. Crystal structures at variable pressures were well optimized on a 6 × 6 × 4 k-point mesh, and VDoS were calculated using the finite displacement method as implemented in the code PHONOPY^67^. The elastic tensors at static conditions were calculated from the linear dependence of stress on the small strain. Owing to the enormous computational cost of calculating the vibrational density of state based on LDA+*U*, we used the 20-atom unit cell for (Mg_0.5_Fe_0.5_)(Si_0.5_Fe_0.5_)O_3_ bridgmanite^43–45^. Similar to previous studies on the elastic properties of Fe-bearing bridgmanite^45,46^, we only report results for aggregate elastic moduli, not individual elastic coefficients. The latter are sensitive to atomic configurations and therefore to supercell size, which can accommodate different configurations for the same composition. The aggregate elastic moduli, *K*_*S*_ and *G*, are quite insensitive to the atomic configuration^68–70^.

The Helmholtz free energy calculated from Eq. (2) within the QHA versus volume was fitted by the isothermal third-order finite strain equation of state, and then we can obtain all thermodynamic properties, such as pressures at different temperatures and volumes. Our results show that (Mg_0.5_Fe_0.5_)(Si_0.5_Fe_0.5_)O_3_ bridgmanite has a larger volume than (Mg_0.5_Fe_0.5_)(Si_0.5_Al_0.5_)O_3_ bridgmanite (Fig. 2). The substitution of Al^3+^ for LS Fe^3+^ in the octahedral site causes a slight decrease of ~1.0% in volume at >80 GPa. Compared to ﻿pristine Bdg (MgSiO_3_), these two Fe^3+^- and Al^3+^-rich species have larger volumes; for example, at 90 GPa and 2000 K, the volumes of (Mg_0.5_Fe_0.5_)(Si_0.5_Al_0.5_)O_3_ and (Mg_0.5_Fe_0.5_)(Si_0.5_Fe_0.5_)O_3_ are 3.5% and 4.4% larger than that of MgSiO_3_, respectively.

Using the equation of states, we transferred volume- and temperature-dependent elasticity into pressure- and temperature-dependent elasticity. The adiabatic bulk modulus *K*_*S*_ and shear modulus *G* can be obtained by computing the Voigt–Reuss–Hill averages^71^ from elastic tensors. Thus, compressional and shear velocities can be calculated from the equations $V_P = \sqrt {(Ks + \frac{4}{3}G)/\rho }$ and $V_S = \sqrt {G/\rho }$ (*ρ* is density). Bulk moduli (*K*_*S*_), shear moduli (*G*), compressional-wave velocity (*V*_*P*_), and shear-wave velocity (*V*_*S*_) are also derived from LDA*+U* calculations as shown in Supplementary Fig. 3. Compared to (Mg_0.5_Fe_0.5_)(Si_0.5_Fe_0.5_)O_3_ Bdg, (Mg_0.5_Fe_0.5_)(Si_0.5_Al_0.5_)O_3_ Bdg has a lower density, similar *K*_*S*_ but much larger *G* at >90 GPa, which results in much higher velocities in (Mg_0.5_Fe_0.5_)(Si_0.5_Al_0.5_)O_3_ Bdg. At 100 GPa and 2000 K, the differences in density, *K*_*S*_*, G, V*_*P*_, and *V*_*S*_ between (Mg_0.5_Fe_0.5_)(Si_0.5_Al_0.5_)O_3_ and (Mg_0.5_Fe_0.5_)(Si_0.5_Fe_0.5_)O_3_ are −10.4%, −0.9%, 19.2%, 8.1%, and 14.8%, respectively. Elastic moduli and velocities almost linearly depend on pressure and temperature after B-site Fe^3+^ spin transition and their first pressure and temperature derivatives are comparable to those of MgSiO_3_ Bdg (Supplementary Table 3).

### Ab initio investigation on the stability of (Mg_0.5_Fe_0.5_)(Si_0.5_Fe_0.5_)O_3_ bridgmanite

Through high-pressure and high-temperature experiments, we find the formation of iron-rich bridgmanite (Mg_0.5_Fe_0.5_)(Si_0.5_Fe_0.5_)O_3_ coexisting with Fe-poor bridgmanite, instead of forming a single phase of (Mg_0.9_Fe_0.1_)(Si_0.9_Fe_0.1_)O_3_ Bdg with homogeneous iron content. In order to check the relative stability of (Mg_0.5_Fe_0.5_)(Si_0.5_Fe_0.5_)O_3_ bridgmanite, we also calculated the formation energy of the decomposition reaction:

(Mg_0.875_Fe_0.125_)(Si_0.875_Fe_0.125_)O_3_ ↔ 3/4MgSiO_3_+1/4(Mg_0.5_Fe_0.5_)(Si_0.5_Fe_0.5_) (4)

The Gibbs free energy of (Mg_1-x_Fe_x_)(Si_1-x_Fe_x_)O_3_ can be expressed as (see Supplementary Materials):

5 $$G_{{\mathrm{HS/LS}}}\left( {P,T} \right) = G_{{\mathrm{HS/lS}}}^{{\mathrm{stat}} + {\mathrm{vib}}}\left( {P,T} \right) + G_{{\mathrm{HS/LS}}}^{{\mathrm{mag}}}\left( {P,T} \right) - {\mathrm{TS}}^{{\mathrm{conf}}}$$

where *S*^conf^ is the configurational entropy (*S*^conf^ = *k*_*B*_*lnM*, *M* is the configuration degeneracy). $G_{{\mathrm{HS/lS}}}^{{\mathrm{stat}} + {\mathrm{vib}}}\left( {P,T} \right)$ and $G_{{\mathrm{HS/LS}}}^{{\mathrm{mag}}}\left( {P,T} \right)$ can be derived from Eqs. (3–6) in Supplementary Materials. Thus, the Gibbs formation free energy of the decomposition reaction for pure HS/LS state can be expressed as:

6 $$\Delta G = \frac{1}{4} \ast \left( {G_{x = 0.5}^{{\mathrm{stat}} + {\mathrm{vib}}} - {\mathrm{TS}}_{x = 0.5}^{{\mathrm{conf}}}} \right) + \frac{3}{4} \ast G_{{\mathrm{MgSiO3}}}^{{\mathrm{stat}} + {\mathrm{vib}}} - (G_{x = 0.125}^{{\mathrm{stat}} + {\mathrm{vib}}} - {\mathrm{TS}}_{x = 0.125}^{{\mathrm{conf}}})$$

We also investigated the disordered substitution of Fe^3+^ in (Mg_0.875_Fe_0.125_)(Si_0.875_Fe_0.125_)O_3_ bridgmanite in a 40-atom cell ($\sqrt 2 \times \sqrt 2 \times 1$ supercell). Similar to the case for (Mg_0.5_Fe_0.5_)(Si_0.5_Fe_0.5_)O_3_ bridgmanite, the initial structure was constructed by ﻿replacing one nearest-neighbor Mg^2+^–Si^4+^ pair with one [Fe^3+^]_Mg_–[Fe^3+^]^Si^ pair. Due to the extremely high computational cost of VDOS calculation using LDA+*U* functional, we did not calculate the VDOS of (Mg_0.875_Fe_0.125_)(Si_0.875_Fe_0.125_)O_3_ bridgmanite. Because (Mg_1-x_Fe_x_)(Si_1-x_Fe_x_)O_3_ bridgmanite with different Fe^3+^ contents have similar structures, here we assume that the vibrational contribution to the Gibbs free energy linearly depends on Fe^3+^ content ($G_{x = 0.125}^{{\mathrm{vib}}} = \frac{1}{4} \ast G_{x = 0.5}^{{\mathrm{vib}}} + \frac{3}{4} \ast G_{{\mathrm{MgSiO3}}}^{{\mathrm{vib}}}$). Under this approximation, Δ*G* can be written as:

7 $$\Delta G = \frac{1}{4} \ast \left( {H_{x = 0.5}^{{\mathrm{stat}}} - {\mathrm{TS}}_{x = 0.5}^{{\mathrm{conf}}}} \right) + \frac{3}{4} \ast H_{{\mathrm{MgSiO3}}}^{{\mathrm{stat}}} - (H_{x = 0.125}^{{\mathrm{stat}}} - {\mathrm{TS}}_{x = 0.125}^{{\mathrm{conf}}})$$

where *H*^stat^ is the internal energy or the Gibbs free energy without the vibrational contribution.

As shown in Supplementary Fig. 2, ΔG is negative at lower-mantle conditions regardless of the spin state of B-site Fe^3+^ in (Mg_1-x_Fe_x_)(Si_1-x_Fe_x_)O_3_ bridgmanite. This implies that the assemblage of (Mg_0.5_Fe_0.5_)(Si_0.5_Fe_0.5_)O_3_ and MgSiO_3_ bridgmanite is more stable than the single-phase (Mg_0.875_Fe_0.125_)(Si_0.875_Fe_0.125_)O_3_ bridgmanite, consistent with our experimental results (Fig. 1). In addition, our LDA+*U* calculations extend the occurrence of this decomposition reaction to the lowermost-mantle conditions, where (Mg_0.5_Fe_0.5_)(Si_0.5_Fe_0.5_)O_3_ bridgmanite is still more stable than (Mg_0.875_Fe_0.125_)(Si_0.875_Fe_0.125_)O_3_ bridgmanite.

In order to check the effect of the exchange-correlation function on the results, we also calculated the enthalpy change of this reaction using the generalized gradient approximation with Hubbard *U* correction (GGA+*U*). *U* values for Fe^3+^ on A and B sites in bridgmanite are 3.3 and 4.5 eV, respectively. The Δ*H* predicted by the GGA+*U* calculations is similar to that from the LDA+*U* calculations (Supplementary Fig. 2), both of which favor the mixed phases over the single phase.

### Thermoelastic models for the estimations of velocity and density heterogeneities

Previous studies^37^ have suggested that the pyrolitic lower mantle that consists of 15% Mg_0.82_Fe_0.18_O ferropericlase (Fp), 78% Mg_0.92_Fe_0.08_SiO_3_ bridgmanite (Fe^2+^-Bdg), and 7% CaSiO_3_ Ca-perovskite (CaPv)) can admirably reproduce the velocity and density profiles of PREM model^72^ for the lower mantle. Combining the thermoelastic properties^20,46,47^ of these three major minerals with our elastic data for LS-(Mg_0.5_Fe_0.5_)(Si_0.5_Fe_0.5_)O_3_ and (Mg_0.5_Fe_0.5_)(Si_0.5_Al_0.5_)O_3_ bridgmanite, we quantify the dependences of velocity and density anomalies on the amount of Fe^3+^-rich Bdg by substituting a certain proportion of Fe^3+^-free Bdg with (Mg_0.5_Fe_0.5_)(Si_0.5_Fe_0.5_)O_3_ bridgmanite. Compared to the pyrolitic composition, the modeling chemical assemblage has identical mineral fractions (15% ferropericlase + 78% Bdg + 7% CaPv) in which a portion of Fe^2+^-bearing Bdg was substituted by (Mg_0.5_Fe_0.5_)(Si_0.5_Fe_0.5_)O_3_ or (Mg_0.5_Fe_0.5_)(Si_0.5_Fe_0.4_Al_0.1_)O_3_ Bdg. In other words, the ferropericlase and Ca-perovskite contents are fixed to 15% and 7%, respectively. We also explore the effect of Fe^2+^ content in the assemblage (noted by the Fe^2+^ content in Fp, *Fe*^*2+*^_*Fp*_) on modeling results because the incorporation of Fe^2+^ into Fp and Bdg decreases their velocities to some extent^46,47^. The Fe–Mg partition coefficient between Fp and Bdg^73^ is used to constrain their Fe^2+^ contents. The modeled ﻿aggregate with enrichment of Fe^3+^-rich Bdg is composed of 15% Mg_1-x_Fe_x_O ferropericlase, 7% CaSiO_3_ Ca-perovskite, Z% (Mg_0.5_Fe_0.5_)(Si_0.5_Fe_0.5_)O_3_ or (Mg_0.5_Fe_0.5_)(Si_0.5_Fe_0.4_Al_0.1_)O_3_ bridgmanite, and (78-Z)% Mg_1-y_Fe_y_SiO_3_ bridgmanite.

The elastic moduli and densities of the aggregate are calculated using:

8 $$\rho = \mathop {\sum}\nolimits_i {f_i\rho _i}$$

9 $$M = \frac{1}{2}\left[ {\mathop {\sum }\limits_i f_iM_i + \left( {\mathop {\sum }\limits_i f_iM_i^{ - 1}} \right)^{ - 1}} \right]$$

where *ρ*_i_, *M*_*i*_, and *f*_*i*_ are the density, bulk modulus (*K*_*S*_) or shear modulus (*G*), and the fraction of the *i*th mineral, respectively. Similarly, the compressional and shear velocities (*V*_*P*_ and *V*_*S*_) were derived from $V_P = \sqrt {(Ks + \frac{4}{3}G)/\rho }$ and $V_S = \sqrt {G/\rho }$, and hence the velocity and density anomalies relative to the pyrolitic lower mantle are estimated with the consideration of temperature anomaly.

### Geodynamic models

We performed thermochemical calculations to study the dynamics of iron-rich materials in the lowermost mantle. The numerical simulations were conducted by solving the nondimensional equations of conservation of mass, momentum, and energy under the Boussinesq approximation. The intrinsic density anomaly is represented by the buoyancy number *B*, which is defined as the ratio between the intrinsic (compositional) density anomaly and the density anomaly caused by thermal expansion, *B* = Δ*ρ*/(*ρα*Δ*T*), where Δ*ρ* is the intrinsic density anomaly for the iron-rich materials compared to the background mantle. We used *α* = 1 × 10^−5^ K^−1^ and Δ*T* = 2500 K in this study.

The whole-mantle dynamics is simulated in a 2D Cartesian box with an aspect ratio of 3:1. The model domain contains 1536 and 512 elements in the horizontal and vertical directions, respectively. Models are basally heated, with the top and bottom having a fixed temperature at *T* = 0 and *T* = 1, respectively. The top and bottom boundaries are both free-flip and the side boundaries are reflective. The viscosity is determined by *η* = *η*_0_exp[0.5−*T*], where *η*_0_ is a viscosity pre-factor, and *A* is the activation energy. Here, *η*_0_ is 1.0 and 30.0 for the upper mantle and the lower mantle, respectively, and *A* = 9.21 which leads to the viscosity changing four orders of magnitude as temperature increases from 0 to 1. Initially, the whole mantle was assumed to be hot with a temperature of *T* = 0.72 (or 1800 K) everywhere, and we introduced a global layer of iron-rich materials in the lowermost 300 km of the mantle.

We performed four cases. Case 1 is the reference case in which the iron-rich materials are 1.2% intrinsically denser (with a buoyancy number of *B* = 0.48) than the background mantle materials and the Rayleigh number, which controls the vigor of mantle convection, is *Ra* = 1 × 10^7^. Cases 2 and 3 have a Rayleigh number of *Ra* = 1 × 10^6^ and *Ra* = 1 × 10^8^, respectively, while other parameters are the same as case 1. In case 4, the iron-rich materials are 1.5% intrinsically denser (with a buoyancy number of *B* = 0.6) than the background mantle materials, and other parameters are the same as case 1.

## Supplementary information

Supplementary Information

Peer Review File

Description of Additional Supplementary Files

Supplementary Data 1

Supplementary Data 2

## Data Availability

The data are available in the main text, the supplementary materials, and from ﻿the corresponding authors.

## Code availability

The open-source Quantum Espresso package used in this study is available at https://www.quantum-espresso.org/.
