# Supplementary material for: Formation of large low shear velocity provinces through the decomposition of oxidized mantle
Source: Nat Commun. 2021 Mar 26;12:1911. doi: 10.1038/s41467-021-22185-1 (PMC7997914; doi:10.1038/s41467-021-22185-1)
Supplement: Supplementary file 1 — Supplementary Information [file 41467_2021_22185_MOESM1_ESM.docx]

**Supplementary Information for "Formation of large low shear velocity provinces through the decomposition of oxidized mantle"**

Wenzhong Wang^1,2,*^, Jiachao Liu^3,*^, Feng Zhu^4^, Mingming Li^5^, Susannah M. Dorfman^3^, Jie Li^4^, Zhongqing Wu^1,6,7,*^

^1^Laboratory of Seismology and Physics of Earth’s Interior, School of Earth and Space Sciences, University of Science and Technology of China, Hefei, China

^2^Department of Earth Sciences, University College London, London WC1E 6BT, United Kingdom

^3^Department of Earth and Environmental Sciences, Michigan State University, East Lansing, MI 48824, USA

^4^Department of Earth and Environmental Sciences, University of Michigan, Ann Arbor, MI 48109, USA

^5^School of Earth and Space Exploration, Arizona State University, Tempe, AZ 85287, USA

^6^National Geophysical Observatory at Mengcheng, University of Science and Technology of China, Hefei, China

^7^CAS Center for Excellence in Comparative Planetology, USTC, Hefei, Anhui 230026, China

*Correspondence should be addressed to Wenzhong Wang (wz30304@mail.ustc.edu.cn), Jiachao Liu (jiacliu09@gmail.com), and Zhongqing Wu (wuzq10@ustc.edu.cn)

**Supplementary Notes**

**1. The fraction of low-spin B-site Fe^3+^ in (Mg_0.5_Fe_0.5_)(Si_0.5_Fe_0.5_)O_3_ bridgmanite**

The B-site Fe^3+^ in (Mg_0.5_Fe_0.5_)(Al_0.5_Fe_0.5_)O_3_ Bdg undergoes a high-spin (HS) state to low-spin (LS) state transition with increasing pressure, while the A-site Fe^3+^ in both compositions maintain the HS state throughout the lower-mantle conditions^1^. The molar Gibbs free energy for the mixed-spin (MS) state can be expressed as:

$G(P, T, n)=nG_{LS}(P, T)+(1-n)G_{HS}(P, T)+G^{mix}(P, T)$ (1)

where $n$ is the low-spin fraction, $G_{HS/LS}$ is the Gibbs free energy of pure HS/LS state, and $G^{mix}$ is the mixing Gibbs free energy. $P$ and $T$ are pressure and temperature, respectively. The Gibbs free energy for pure HS/LS state can be expressed as:

$G_{HS/LS}(P, T)=G_{HS/LS}^{stat+vib}(P, T)+G_{HS/LS}^{mag}(P, T)$ (2)

where $G_{HS/LS}^{stat+vib}$ is the part of Gibbs free energy that includes the internal energy and the vibrational contribution, and $G_{HS/LS}^{mag}$ is the magnetic contribution to Gibbs free energy.

The first term, $G_{HS/LS}^{stat+vib}$, is given by:

$G_{HS/LS}^{stat+vib}(V, T)=F_{HS/LS}(V, T)+PV$ (3)

where $F_{HS/LS}$ is the Helmholtz free energy of pure HS/LS state. Within the quasi-harmonic approximation (QHA), $F_{HS/LS}(V, T)$ can be expressed as:

$$F_{HS/LS}\left( V,T \right)=U_{HS/LS}\left( V \right)+\frac{1}{2}\sum_{q,m} \hbar\omega_{q,m}^{HS/LS}\left( V \right)+k_{B}T\sum_{q,m} ln(1-exp(-\frac{\hbar\omega_{q,m}^{HS/LS}\left( V \right)}{k_{B}T}))$$

(4)

where $U_{HS/LS}$ is the static energy at the equilibrium volume $V$*,* $\hbar$ and $k_{B}$ are Planck and Boltzmann constants, and $\omega_{q,m}^{HS/LS}$ is the vibrational frequency of the *i^th^* mode along the wave vector $q$. Thus, $G_{HS/LS}^{stat+vib}(V, T)$ can be calculated from the internal energy and vibrational density of state of pure HS/LS state

The second term, $G_{HS/LS}^{mag}$, is given by:

$G_{HS}^{mag}(V, T)=-xk_{B}Tln[m_{HS}(S_{HS}+1)\times m_{HS}(S_{HS}+1)]$ (5)

$G_{LS}^{mag}(V, T)=-xk_{B}Tln[m_{HS}(S_{HS}+1)\times m_{LS}(S_{LS}+1)]$ (6)

where $m_{HS/LS}$ and $S_{HS/LS}$ are the orbital degeneracy and total spin of Fe^3+^ in HS and LS state, respectively. $x$ is the Fe_2_O_3_ molar concentration. Thus, $G_{HS/LS}(V, T)$ can be calculated from $G_{HS/LS}^{stat+vib}(V, T)$ and $G_{HS/LS}^{mag}(V, T)$, and $G_{HS/LS}(P, T)$ can be converted from $G_{HS/LS}(V, T)$ using the equation of state.

The mixing Gibbs free energy, $G^{mix}(P, T)$, can be written as:

$G^{mix}(P, T)=k_{B}Tx[nln(n)+(1-n)ln(1-n)]$ (7)

By Minimizing the molar Gibbs free energy for MS state with respect to the LS fraction, the pressure- and temperature-dependent LS fraction can be expressed as:

$n(P, T)=\frac{1}{1+\frac{m_{HS}({2S}_{HS}+1)}{m_{LS}(2S_{LS}+1)}e^{\frac{{\Delta G}_{LS-HS}^{stat+vib}}{k_{B}Tx}}}$ (8)

where ${\Delta G}_{LS-HS}^{stat+vib}(P, T)$=$G_{LS}^{stat+vib}(P, T)-G_{HS}^{stat+vib}(P, T)$.

**2. The elasticity of mixed-spin (Mg_0.5_Fe_0.5_)(Si_0.5_Fe_0.5_)O_3_ bridgmanite**

Similarly, the LS fraction under stress $\sigma$ can be calculated from the molar Gibbs free energy of pure HS/LS state under stress $\sigma$. Therefore, the LS fraction can be rewritten as:

$n(P, T, \sigma)=\frac{1}{1+\frac{m_{HS}({2S}_{HS}+1)}{m_{LS}(2S_{LS}+1)}e^{\frac{{\Delta G}_{LS-HS}^{stat+vib}}{k_{B}Tx}}}$ (9)

where ${\Delta G}_{LS-HS}^{stat+vib}(P, T, \sigma)$=$G_{LS}^{stat+vib}(P, T, \sigma)-G_{HS}^{stat+vib}(P, T, \sigma)$. The elastic compliance $S_{ij}$ of the mixed-spin (Mg_0.5_Fe_0.5_)(Si_0.5_Fe_0.5_)O_3_ bridgmanite are given by:

$S_{ij}(P, T, \sigma)=-\frac{1}{V}\frac{\partial^{2}G(P, T, \sigma)}{\partial\sigma_{i}\partial\sigma_{j}}$ (10)

Following Eq. (1), the derivative of $G(P, T, \sigma)$ with respect to $\sigma$ can be expressed as:

$\frac{\partial G}{\partial\sigma_{i}}=n\frac{\partial G_{LS}}{\partial\sigma_{i}}+(1-n)\frac{\partial G_{HS}}{\partial\sigma_{i}}+(G_{LS}-G_{HS})\frac{\partial n}{\partial\sigma_{i}}+\frac{\partial G^{mix}}{\partial\sigma_{i}}$ (11)

At equilibrium,

$\frac{\partial G}{\partial n}=[(G_{LS}-G_{HS})\frac{\partial n}{\partial\sigma_{i}}+\frac{\partial G^{mix}}{\partial\sigma_{i}}] \frac{\partial\sigma_{i}}{\partial n}=0$ (12)

Because $\frac{\partial\sigma_{i}}{\partial n}\neq0$, we can obtain:

$(G_{LS}-G_{HS})\frac{\partial n}{\partial\sigma_{i}}+\frac{\partial G^{mix}}{\partial\sigma_{i}}=0$ (13)

Thus, the elastic compliance can be written as:

$S_{ij}V=nS_{ij}^{LS}V_{LS}+(1-n)S_{ij}^{HS}V_{HS}+(\frac{\partial G_{HS}}{\partial\sigma_{i}}-\frac{\partial G_{LS}}{\partial\sigma_{i}})\frac{\partial n}{\partial\sigma_{j}}$ (14)

Here volume $V$ in MS state is calculated using $V=nV_{LS}+(1-n)V_{HS}$. For $i, j=1, 2, 3$, using $\frac{\partial}{\partial\sigma_{i}}=\frac{\partial}{\partial P}\frac{\partial P}{\partial\sigma_{i}}=\frac{1}{3}\frac{\partial}{\partial P}$, we obtain

$S_{ij}V=nS_{ij}^{LS}V_{LS}+(1-n)S_{ij}^{HS}V_{HS}+\frac{1}{9}(V_{HS}-V_{LS})\frac{\partial n}{\partial P}$ (15)

For $i, j=4, 5, 6$, as $\frac{\partial n}{\partial\sigma_{i}}\approx0$ for orthorhombic systems, the elastic compliances are:

$S_{ij}V=nS_{ij}^{LS}V_{LS}+(1-n)S_{ij}^{HS}V_{HS}$ (16)

Finally, the elastic constants, $C_{ij}$, can be derived from the inverse of $S_{ij}$, and the adiabatic bulk modulus *K_S_* and shear modulus *G* can be obtained by computing the Voigt-Reuss-Hill averages^2^ from $C_{ij}$.

**Supplementary Table 1**. The summary of the pressure-temperature-duration conditions and run products of the multi-anvil experiments for 10 mol% Fe_2_O_3_-90 mol% MgSiO_3_ system.

| Experiments | Pressure | Temperature | Duration | Fe-rich phase | Fe-poor phase |
| --- | --- | --- | --- | --- | --- |
| # | GPa | K | hours |  |  |
| D080716 | 10 | 1573 | 10 | Fe_1.98(2)_O_3_ | Mg_0.97(3)_Si_1.00(1)_O_3_ |
| D100616 | 15 | 1773 | 7 | (Mg_0.33(7)_Fe_0.49(4)_)(Si_0.87(2)_Fe_0.13(4)_)O_3_ | Fe_0.17(1)_Mg_1.74(2)_Si_1.00(2)_O_4_ |
| D100416 | 20 | 1873 | 6 | (Mg_0.56(5)_Fe_0.44(5)_)(Si_0.55(3)_Fe_0.45(5)_)O_3_ | (Mg_0.89(6)_Fe_0.09(2)_)(Si_0.94(4)_Fe_0.06(2)_)O_3_ |
| D052116 | 24 | 1873 | 8 | (Mg_0.52(1)_Fe_0.48(1)_)(Si_0.53(1)_Fe_0.47(1)_)O_3_ | (Mg_0.91(3)_Fe_0.08(1)_)(Si_0.95(2)_Fe_0.05(1)_)O_3_ |
| D062116 | 24 | 1873 | 24 | (Mg_0.55(4)_Fe_0.45(3)_)(Si_0.54(3)_Fe_0.46(3)_)O3 | (Mg_0.92(1)_Fe_0.08(1)_)(Si_0.96(1)_Fe_0.04(1)_)O_3_ |

**Supplementary Table 2**. The first-order derivatives of elastic moduli and wave velocities with respect to pressure and temperature for (Mg_0.5_Fe_0.5_)(Si_0.5_Fe_0.5_)O_3_, (Mg_0.5_Fe_0.5_)(Si_0.5_Al_0.5_)O_3_ bridgmanite compared with those for MgSiO_3_ bridgmanite.

| Bdg | *∂K_S_/∂P* | *∂K_S_/∂T* MPa/K | *∂G/∂P* | *∂G/∂T* MPa/K | *∂V_P_/∂P* km·s^-1^·GPa^-1^ | *∂V_P_/∂T* km·s^-1^·K^-1^ | *∂V_S_/∂P*  km·s^-1^·GPa^-1^ | *∂V_S_/∂T* km·s^-1^·K^-1^ |
| --- | --- | --- | --- | --- | --- | --- | --- | --- |
| (Mg_0.5_Fe_0.5_)(Si_0.5_Fe_0.5_)O_3_ | 3.47 | -20.7 | 0.74 | -11.4 | 0.0191 | -1.71×10^-4^ | 0.0054 | -1.24×10^-4^ |
| (Mg_0.5_Fe_0.5_)(Si_0.5_Al_0.5_)O_3_ | 3.21 | -18.3 | 0.84 | -13.1 | 0.0188 | -1.85×10^-4^ | 0.0058 | -1.40×10^-4^ |
| MgSiO_3_ | 3.27 | -14.6 | 1.04 | -12.5 | 0.0201 | -1.48×10^-4^ | 0.0070 | -1.23×10^-4^ |

The pressure and temperature used to obtain these parameters for (Mg_0.5_Fe_0.5_)(Si_0.5_Al_0.5_)O_3_ and MgSiO_3_ bridgmanite^3^ range from 70 GPa to 130 GPa and 300 K to 3000 K, respectively. For (Mg_0.5_Fe_0.5_)(Si_0.5_Fe_0.5_)O_3_ bridgmanite, we only focus on the P-T ranges in which B-site Fe^3+^ is completely under LS state.


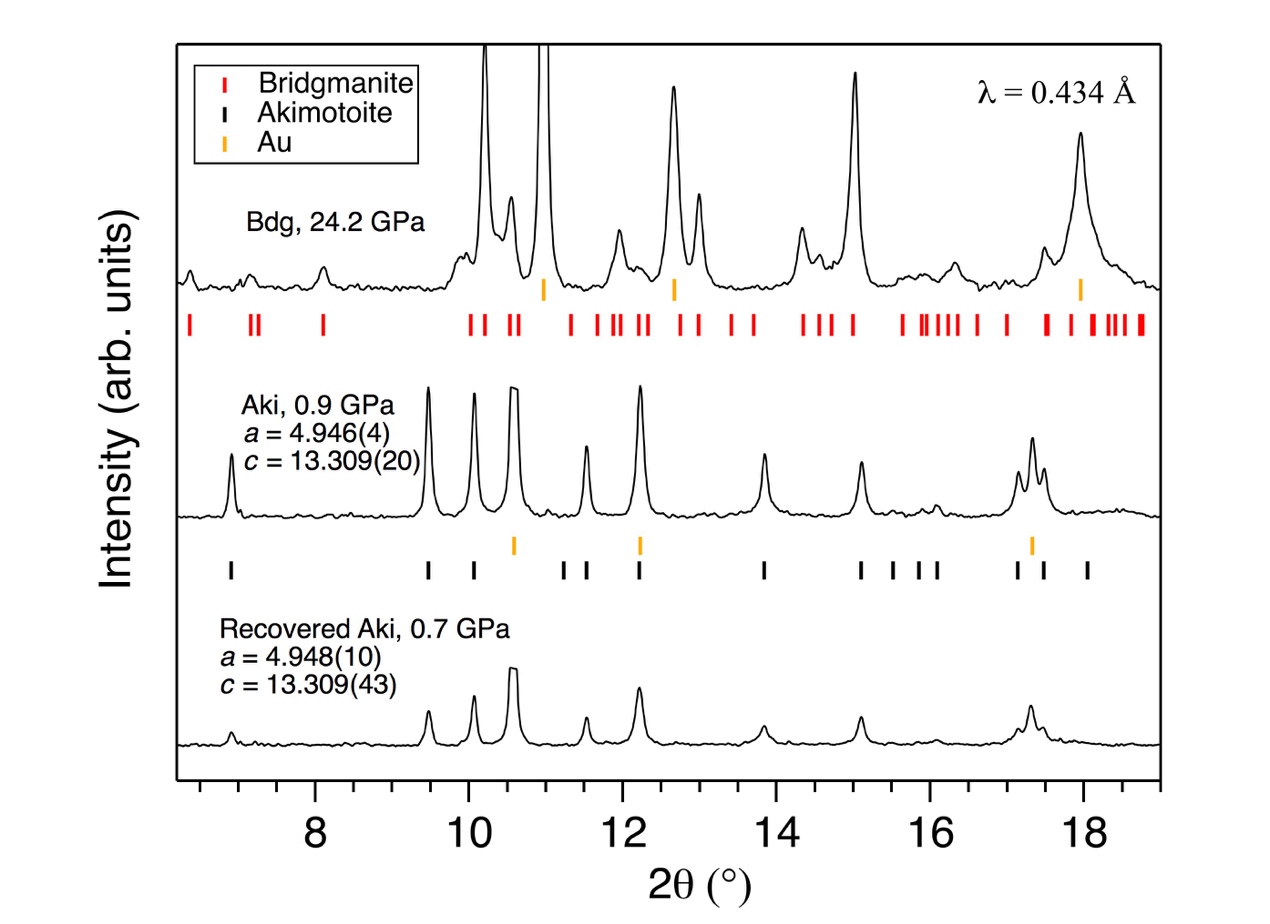


**Supplementary Figure 1.** Measured XRD data at 300 K for (Mg_0.46_Fe^3+^_0.53_)(Si_0.49_Fe^3+^_0.51_)O_3_ akimotoite at 0.9 GPa and the transformed bridgmanite at ~ 24.2 GPa together with the recovered akimotoite at 0.7 GPa. The expected peak positions for akimotoite, bridgmanite and Au calibrant are shown as black, red and yellow ticks, respectively. The XRD intensity of Au (111) peak is truncated for clarity. The fitted lattice parameters for akimotoite spectra are listed aside the corresponding spectra.


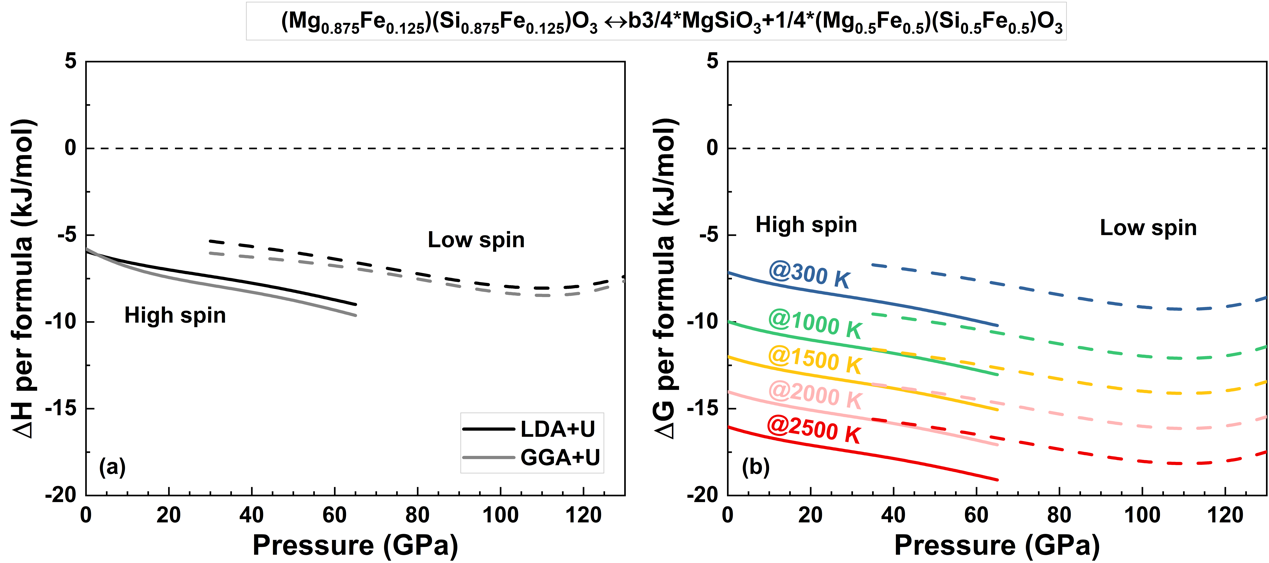


**Supplementary Figure 2. Enthalpy change (ΔH) and Gibbs formation free energy (ΔG) of the decomposition of (Mg_0.875_Fe_0.125_)(Si_0.875_Fe_0.125_)O_3_ into (Mg_0.5_Fe_0.5_)(Si_0.5_Fe_0.5_)O_3_ and MgSiO_3_ bridgmanite.** The calculated results demonstrate that ΔG based on LDA+U is negative at lower-mantle conditions regardless of the spin state of B-site Fe^3+^ in (Mg_1-x_Fe_x_)(Si_1-x_Fe_x_)O_3_ bridgmanite, suggesting that the assemblage of (Mg_0.5_Fe_0.5_)(Si_0.5_Fe_0.5_)O_3_ and MgSiO_3_ bridgmanite is more stable than single-phase (Mg_0.875_Fe_0.125_)(Si_0.875_Fe_0.125_)O_3_ bridgmanite.


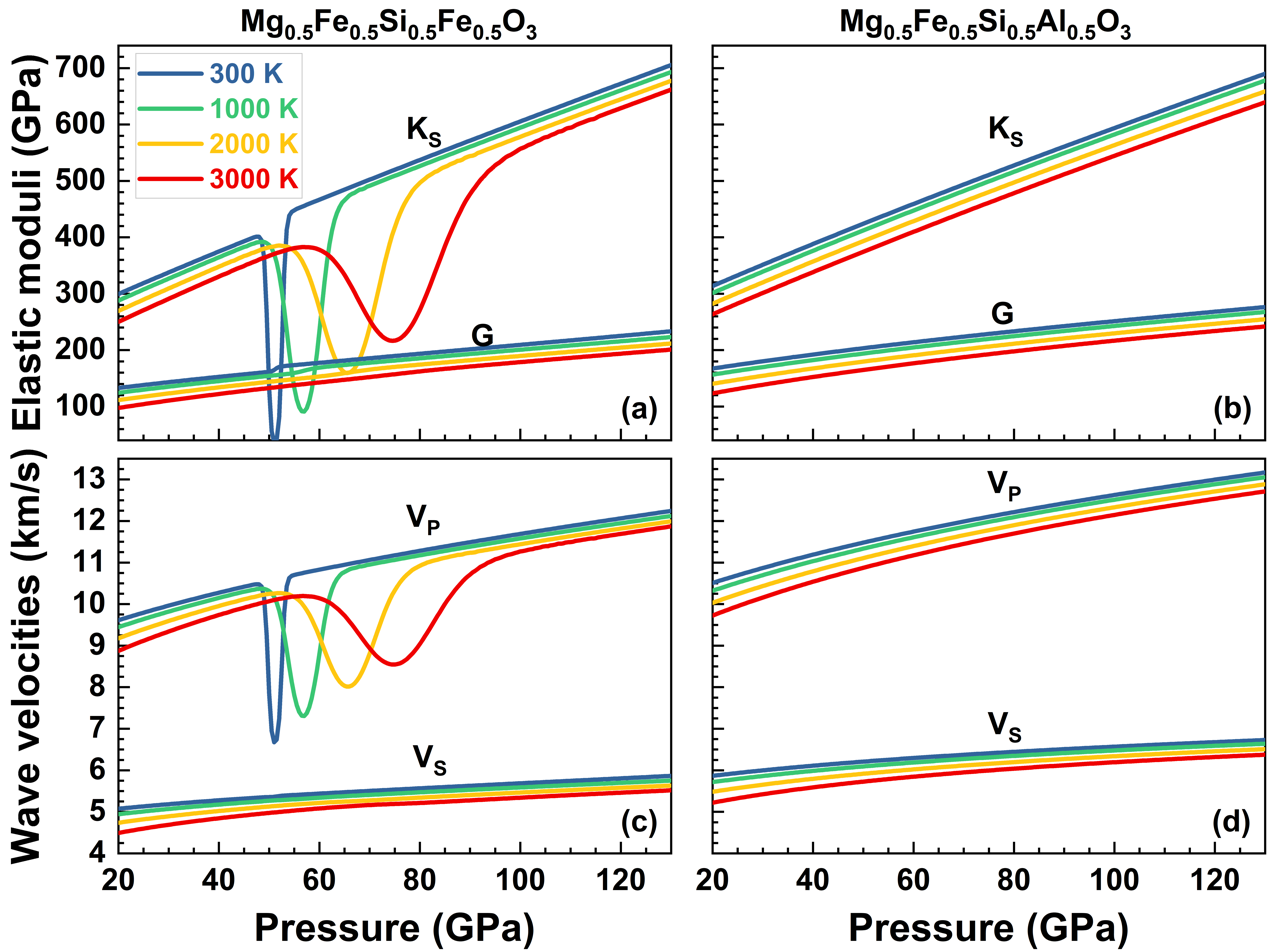


**Supplementary Figure 3.** **Bulk moduli and wave velocities of (Mg_0.5_Fe_0.5_)(Si_0.5_Fe_0.5_)O_3_ and (Mg_0.5_Fe_0.5_)(Si_0.5_Al_0.5_)O_3_ bridgmanite.** (a) (b) bulk and shear moduli (K_S_ and G), (c) (d) compressional and shear wave velocities (V_P_ and V_S_) of (Mg_0.5_Fe_0.5_)(Si_0.5_Fe_0.5_)O_3_ and (Mg_0.5_Fe_0.5_)(Si_0.5_Al_0.5_)O_3_ bridgmanite at different pressures and temperatures.


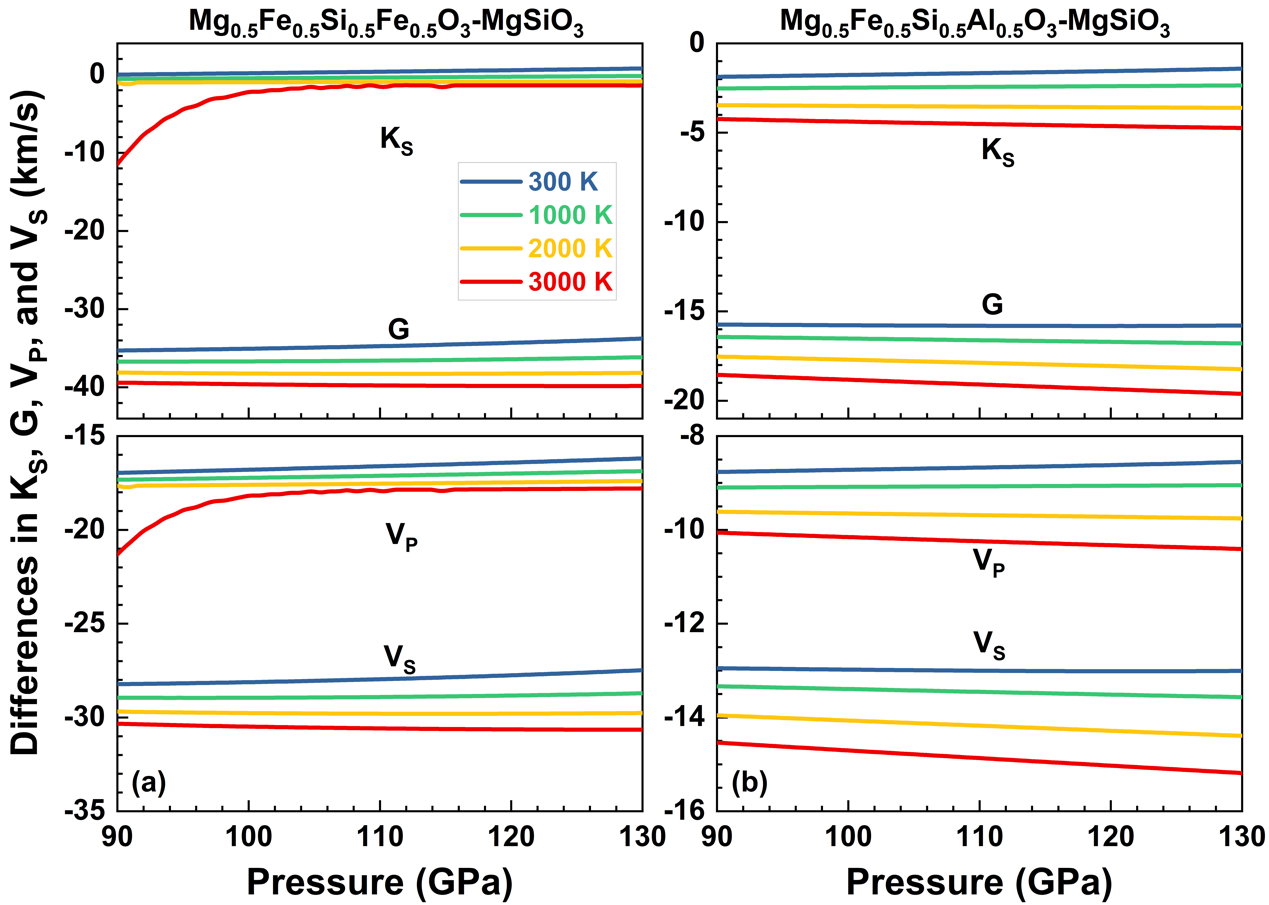


**Supplementary Figure 4.** **The differences in elastic moduli and wave velocities between Fe^3+^-rich and MgSiO_3_ bridgmanite.** The K_S_, G, density, V_P_, and V_S_ between (a) (Mg_0.5_Fe_0.5_)(Si_0.5_Fe_0.5_)O_3_ and MgSiO_3_, (b) (Mg_0.5_Fe_0.5_)(Si_0.5_Al_0.5_)O_3_ and MgSiO_3_. Thermoelastic properties of (Mg_0.5_Fe_0.5_)(Si_0.5_Fe_0.5_)O_3_ and (Mg_0.5_Fe_0.5_)(Si_0.5_Al_0.5_)O_3_ bridgmanite are shown in Supplementary Figure 4. The elastic properties of MgSiO_3_ were calculated using the same method in Shukla et al. (2016)^3^.


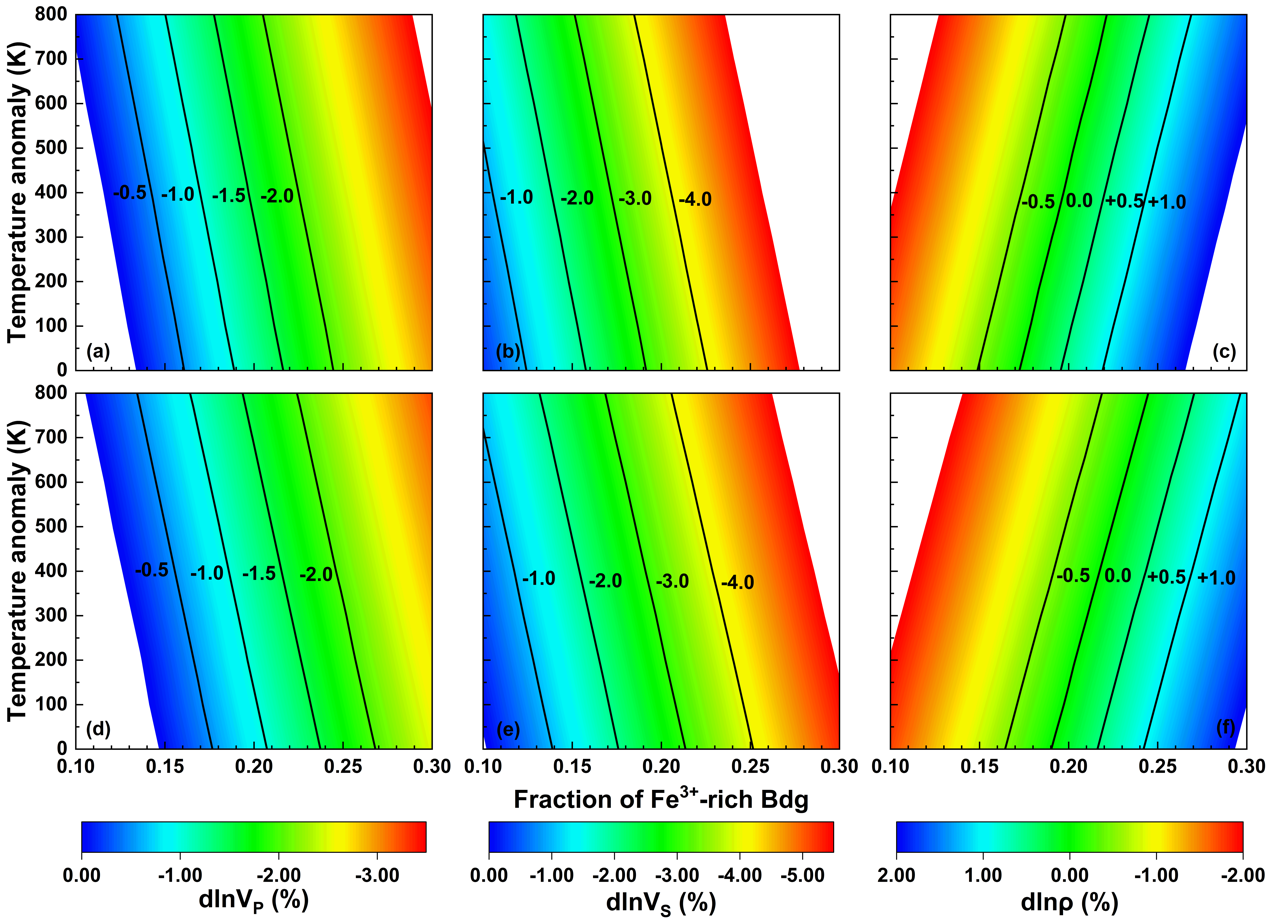


**Supplementary Figure 5.** **Velocity and density anomalies caused by the enrichment of Fe^3+^-rich bridgmanite relative to the pyrolitic composition at 110 GPa without the presence of Fe^2+^.** **(a) (b) (c)** the enrichment of (Mg_0.5_Fe_0.5_)(Si_0.5_Fe_0.5_)O_3_ Bdg; **(d) (e) (f)** the enrichment of (Mg_0.5_Fe_0.5_)(Si_0.5_Fe_0.4_Al_0.1_)O_3_ Bdg if 5% Al_2_O_3_ would be incorporated into (Mg_0.5_Fe_0.5_)(Si_0.5_Fe_0.5_)O_3_. Velocities and density of the pyrolitic lower mantle are calculated using the best-fit composition of the lower mantle (15% Mg_0.82_Fe_0.18_O Ferropericlase (Fp), 78% Mg_0.92_Fe_0.08_SiO_3_ bridgmanite (Fe^2+^-Bdg), and 7% CaSiO_3_ Ca-perovskite (CaPv))^4^. The modeling chemical assemblage has pyrolitic mineral fractions (15% ferropericlase (Fp) + 78% Bdg + 7% CaPv) in which a portion of Fe^2+^-bearing Bdg was substituted by (Mg_0.5_Fe_0.5_)(Si_0.5_Fe_0.5_)O_3_ Bdg. The Fe^2+^ contents in Fp and Bdg are 0 mol%. The temperature anomaly of 0 K refers to the normal mantle temperature from Brown and Shankland (1981)^5^. Data sources for elasticity at high pressure and temperature: Fp, ref. ^6^; Fe^2+^-Bdg, ref. ^3^; Ca-Pv, ref. ^7^.

**Supplementary References**

1. Hsu, H., Blaha, P., Cococcioni, M. & Wentzcovitch, R. M. Spin-State Crossover and Hyperfine Interactions of Ferric Iron in MgSiO_3_ Perovskite. *Phys. Rev. Lett.* **106**, 118501 (2011).

2. Hill, R. The Elastic Behaviour of a Crystalline Aggregate. *Proc. Phys. Soc. Sect. A* **65**, 349–354 (1952).

3. Shukla, G. *et al.* Thermoelasticity of Fe^2+^-bearing bridgmanite. *Geophys. Res. Lett.* **42**, 1741–1749 (2015).

4. Wu, Z. Velocity structure and composition of the lower mantle with spin crossover in ferropericlase. *J. Geophys. Res. Solid Earth* **121**, 2304–2314 (2016).

5. Brown, J. M. & Shankland, T. J. Thermodynamic parameters in the Earth as determined from seismic profiles. *Geophys. J. Int.* **66**, 579–596 (1981).

6. Wu, Z., Justo, J. F. & Wentzcovitch, R. M. Elastic Anomalies in a Spin-Crossover System: Ferropericlase at Lower Mantle Conditions. *Phys. Rev. Lett.* **110**, 228501 (2013).

7. Kawai, K. & Tsuchiya, T. Small shear modulus of cubic CaSiO_3_ perovskite. *Geophys. Res. Lett.* **42**, 2718–2726 (2015).
