# Supplementary material for: Formation of large low shear velocity provinces through the decomposition of oxidized mantle
Source: Nat Commun. 2021 Mar 26;12:1911. doi: 10.1038/s41467-021-22185-1 (PMC7997914; doi:10.1038/s41467-021-22185-1)
Supplement: Supplementary file 3 — Description of Additional Supplementary Files [file 41467_2021_22185_MOESM3_ESM.pdf]

Description of Additional Supplementary Files

File name: Supplementary Data 1

Description: Calculated volume-pressure relationship at static conditions

File name: Supplementary Data 2

Description: Calculated full elasticity results at 2000 K
